# Tyrosine 705 Phosphorylation of STAT3 Is Associated with Phenotype Severity in TGF*β*1 Transgenic Mice

**DOI:** 10.1155/2015/843743

**Published:** 2015-08-24

**Authors:** Eleonora Guadagnin, Jigna Narola, Carsten G. Bönnemann, Yi-Wen Chen

**Affiliations:** ^1^Research Center for Genetic Medicine, Children's National Medical Center, 111 Michigan Avenue, NW, Washington, DC 20010, USA; ^2^Neuromuscular and Neurogenetic Disorders of Childhood Section, Neurogenetics Branch, National Institute of Neurological Disorders and Stroke, National Institutes of Health, 35 Convent Drive, Building 35, Room 2A116, Bethesda, MD 20892, USA; ^3^Department of Integrative Systems Biology and Department of Pediatrics, George Washington University, 2121 I Street Northwest, Washington, DC 20052, USA

## Abstract

Transforming growth factor beta 1 (TGF*β*1) is a key player in skeletal muscle degenerative and regenerative processes. We previously showed that conditionally overexpressing TGF*β*1 in skeletal muscles caused myofiber atrophy and endomysial fibrosis in mice. However, the disease severity varied significantly among individual mice. While 40% of mice developed severe muscle pathology and lost body weight within 2 weeks of TGF*β*1 transgene induction in muscles, the rest showed milder or no phenotype. This study aims at determining whether signal transducer and activator of transcription 3 (STAT3) plays a role in the phenotypic difference and whether it can be activated by TGF*β*1 directly in muscle cells. Our results show that while total STAT3 was not differentially expressed between the two groups of mice, there was significantly higher pSTAT3 (Tyr705) in the muscles of the mice with severe phenotype. Immunohistochemistry showed that pSTAT3 (Tyr705) was localized in approximately 50% of the nuclei of the muscles. We further showed that TGF*β*1 induced Tyr705 phosphorylation of STAT3 in C2C12 cells within 30 minutes of treatment while total STAT3 was not affected. Our findings suggest that TGF*β*1 alone can induce Tyr705 phosphorylation of STAT3 in skeletal muscle cells and contribute to disease severity in transgenic TGF*β*1 mice.

## 1. Introduction

TGF*β*1 belongs to the TGF*β* superfamily and has been shown to regulate a wide variety of biological processes, including promotion of apoptosis, inhibition of cell growth, and induction of cell differentiation, migration, and extracellular matrix (ECM) deposition [1, 2]. Several studies showed that persistent expression and activation of TGF*β*1 act as negative regulator of muscle repair by inducing apoptosis in myoblasts, suppressing muscle differentiation, and causing fibrosis in the muscles [3–5]. TGF*β*1 is believed to be responsible for the ECM deposition in skeletal muscle [6–9], which leads to endomysial and perimysial fibrosis in muscular dystrophies, including Duchenne muscular dystrophy and congenital muscular dystrophies [10–12]. Using animal models, we and others demonstrated that TGF*β*1 alone can cause muscle atrophy and fibrosis* in vivo* [5, 13, 14]. However, TGF*β*1 is also recognized to play critical roles in muscle regeneration process by recruiting macrophages to clean up the damaged tissues after muscle injury and in muscle diseases [15].

The signal transducer and activator of transcription (STAT) family are composed of latent cytoplasmic proteins with a dual molecular role: signal transducer and transcription activator [16, 17]. One member of the STAT family, STAT3, is expressed in most of tissue types and responds mainly to IL-6, IL-10, and EGF signals [18, 19]. Phosphorylation of specific receptor tyrosine residue (Tyr705 or Ser727) in response to ligand stimulation determines the activities of STAT3. Tyr705 phosphorylation of latent cytoplasmic STAT3 promotes STAT3 homodimerization or heterodimerization with other STATs, which leads to nucleus translocation and DNA binding. Ser727 phosphorylation takes place at the C-terminal transactivation domain of STAT3 and allows maximal activation of transcription of its target genes [20]. Within hours, STAT3 is exported back to the cytoplasm and the signaling cascade is terminated [21–23]. Previous studies showed that TGF*β*1 directly activates STAT3 in other cell types, including proximal tubular cells, T-cells, and pancreas [24–26]. One study showed that STAT3 activation by TGF*β*1 plays a major role in the pathological connective tissue deposition in liver via the activation of connective tissue growth factor (CTGF) in hepatic cells [27]. The same study showed that STAT3 inhibition was sufficient to prevent CTGF induction and fibrosis by TGF*β*1. While it is known that STAT3 activation in response to IL-6 stimulation plays major roles in modulating muscle mass, to date there is no direct evidence that TGF*β*1 activates STAT3 in skeletal muscle cells.

To study the effects of TGF*β*1 on muscle fibrosis and atrophy, we generated a tet-repressible muscle-specific TGF*β*1 transgenic mouse model [5]. In this model, withdrawal of oral doxycycline induces the expression of TGF*β*1 transgene. The study showed that TGF*β*1 overexpression in skeletal muscles causes muscle atrophy and endomysial fibrosis. Interestingly, we observed that a subgroup of the TGF*β*1 transgenic mice showed more severe muscle weight loss while the rest exhibited milder pathology. The size of the myofibers was significantly smaller and the endomysial fibrosis was significantly higher in the subgroup with severe phenotypes, suggesting that activation of additional signaling pathways leads to more severe phenotypes. In this study, we investigated whether the STAT3 and phosphorylation of the protein in the mice were associated with more severe phenotype. In addition, we conducted an* in vitro* study using C2C12 myoblasts to determine whether TGF*β*1 can activate STAT3 in muscle cells.

## 2. Materials and Methods

### 2.1. Mouse Model and Muscle Collection

All muscle samples used in this were collected as described previously [5]. Briefly, the tet-repressible muscle-specific TGF-*β*1 transgenic mice (TRE-TGF-*β*1/mCK-tTA) were generated by crossing two transgenic mouse lines (TRE-TGF-*β*1 and mCK-tTA). The TRE-TGF-*β*1 line carries a porcine TGF-*β*1 cDNA containing a double mutation where cysteines at positions 223 and 225 are converted to serines, which is regulated by the tetO recognition element (TRE). The mCK-tTA line carries a construct containing the tetracycline-controlled transactivator (tTA) protein driven by a muscle-specific creatine kinase promoter (mCK). The presence of doxycycline in cells inhibits binding of tTA to the TRE and blocks TGF-*β*1 transgene expression. After crossbreeding the TRE-TGF-*β*1 and mCK-tTA lines, the pregnant female mice received drinking water with doxycycline (200 *μ*g/mL in 5.0% sucrose) in order to suppress the TGF-*β*1 transgene expression in the pups in utero. After weaning, all pups were maintained on water treated with doxycycline until the transgene was induced. In this study, doxycycline was removed from water to induce transgene expression in the TRE-TGF*β*1/mCK-tTA mice when the mice were 6 weeks old. The muscles were collected 2 weeks after the TGF*β*1 transgene was induced. Littermates with only one of the transgenes, which do not express TGF*β*1 were used as controls.

### 2.2. Immunoblotting

Vastus lateralis muscles were sectioned with a Leica CM 1900 cryostat (Walldorf, Baden-Wurttemberg, Germany). Thirty 10 *μ*m cryosections were lysed in 50 *μ*L of RIPA buffer (0.1% SDS, 1% NP40, 0.5% sodium deoxycholate, 150 mM sodium chloride, and 50 mM TrisHCl pH 7.5) for 30 minutes on ice, with protease inhibitor cocktail (Complete, Roche, Mannheim, Germany) as well as phosphatase inhibitor cocktail (PhosStop, Roche, Mannheim, Germany). At the end of the incubation, the cell extracts were centrifuged for 10 minutes (12,000 g) at 4°C. The amount of protein was calculated using the Quick Start Bradford Protein Assay Kit 1 (Bio Rad Laboratories, Hercules, CA). Then 30 *μ*g of protein in NuPAGE LDS Sample Buffer (Life Technologies, Grand Island, NY) and NuPAGE Sample Reducing Agent (Life Technologies, Grand Island, NY) was loaded to SDS-PAGE gel for immunoblotting analysis. The primary antibodies used were pSTAT3 (Y705, 1 : 1000; Cell Signaling Technology, Danvers, MA), pSTAT3 (S727, 1 : 1000; Cell Signaling Technology, Danvers, MA), and Total STAT3 (1 : 1000; Cell Signaling Technology, Danvers, MA). Bound antibodies were detected using ECL reagents. The results were normalized to GAPDH (1 : 5000; Millipore, Billerica, MA). Band intensity was evaluated by densitometry analysis, normalized to its total content, and reported as fold increase relative to respective control set as 1.

### 2.3. Immunofluorescence Staining

To detect pSTAT3 (Tyr705), muscle sections of 5 *μ*m were fixed in 4% paraformaldehyde, washed 3 times in 1x PBS, permeabilized with 0.5% Triton X-100 for 10 minutes at room temperature, and blocked with 5% goat serum. The slides were incubated overnight at 4°C with the primary antibody against pSTAT3 Tyr705 (1 : 100 diluted in 5% goat serum). Secondary antibody only was used as the negative control. After 3 washes in PBS for 15 minutes each, the slides were incubated with the secondary antibody Alexa Fluor 680 Donkey Anti-Rabbit IgG (Life Technlogies, Grand Island, NY) for 1 hour at room temperature and then washed again 3 times in 1x PBS. Finally, the slides were mounted with the appropriate mounting medium (ProLong Gold Antifade Reagent with DAPI, Molecular Porbes, Life Technologies, Grand Island, NY). Images of the tissue sections (20x, 40x) were taken using Nikon Eclipse E800 microscope (Nikon, Chiyoda-ku, Tokyo, Japan), RT slider camera (Diagnostic Instrument, Sterling Height, MI), and SPOT advanced software.

### 2.4. Cell Culture and Treatment

Murine C2C12 myoblasts were cultured in DMEM (Life Technologies, Grand Island, NY) supplemented with 10% fetal bovine serum, 2 mM L-glutamine, and 100 U/mL penicillin/streptomycin at 37°C in 5% CO_2_. Cells were seeded in 6-well plates, and when they were 70% confluent, they were induced to differentiate with DMEM supplemented with 5% horse serum, 2 mM L-glutamine, and 100 U/mL penicillin/streptomycin at 37°C in 5% CO_2_. TGF*β*1 (R&D SYSTEMS, Minneapolis, MN) was reconstituted at 20 *μ*g/mL in sterile 4 mM HCl containing 1 mg/mL BSA, according to the manufacturer's instructions. C2C12 were then treated with TGF*β*1 10 ng/mL after 7 days of differentiation for 30 minutes, 2 hours, and 24 hours. The cells were harvested and lysed in 30 *μ*L of RIPA buffer for immunoblotting.

### 2.5. Statistical Analysis

Data are shown as mean ± SEM. The Kruskal-Wallis test was used for determining statistical significance among different groups of mice. Values of *p* < 0.05 were considered significant. Student's *t*-test was used for determining statistical significance in treated cells. Values of *p* < 0.05 were considered significant.

## 3. Results

### 3.1. Tyr705 Phosphorylation of STAT3 Is Associated with the Severe Phenotypes Induced by TGF*β*1

After the TGF*β*1 transgene was induced for two weeks, approximately 40% of mice developed severe phenotypes, including early body weight loss and severe myofiber atrophy and fibrosis [5]. In the study, the mice in this group were defined as mice with early onset (EO). The rest of mice were grouped into the late onset (LO) group. To determine whether STAT3 activation, which is known to be involved in muscle atrophy induced by IL-6, is involved in the variation of phenotypic presentations, we first examined the protein expression of total STAT3 as well as two phosphorylated STAT3, pSTAT3 (Tyr705), and pSTAT3 (Ser727), in muscles collected from the two groups of mice. Littermates of these mice, which did not express TGF*β*1, were used as baseline control.

**Figure 1:**
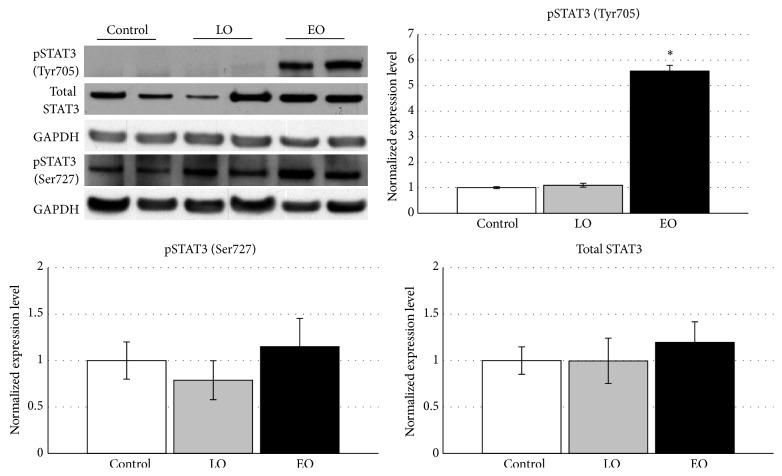
STAT3 phosphorylation level following TGF*β*1 overexpression. Representative western blot of vastus lateralis muscle protein samples of controls (*n* = 6), LO (*n* = 6), and EO (*n* = 6), with anti-phopsho-STAT3 (Tyr705), anti-phospho-STAT3 (Ser727), anti-total-STAT3, and anti-GAPDH. Graphs show normalized expression values of pSTAT3 (Tyr705), pSTAT3 (Ser727), and total STAT3 normalized to GAPDH ± SEM. All the values are normalized for the average of the control. ∗ indicates *p* < 0.05.

Immunoblotting analysis showed that while the total STAT3 was not significantly different among the EO, LO, and control groups, pSTAT3 (Tyr705) was significantly induced in the muscles of EO mice. No pSTAT3 (Tyr705) was detected in the control or LO mice (Figure 1). The expression of pSTAT3 (Ser727) was observed in muscles of all 3 groups but no significant difference among them. Variations of expression levels of total STAT3 and pSTAT3 (Ser727) were observed among different samples in all three groups. However, no correlation between the total STAT3 and pSTAT3 (Ser727) was observed.

### 3.2. pSTAT3 (Tyr705) Is Localized in the Nucleus of Myofibers in the TGF*β*1 Mice with Severe Phenotype

**Figure 2:**
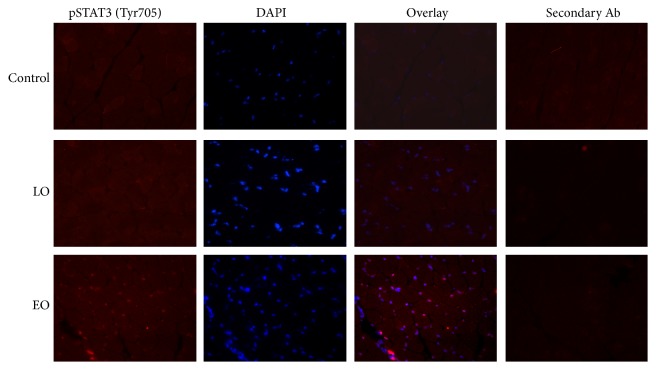
Phospho-STAT3 (Tyr705) localization. Immunofluorescence of phospho-STAT3 (Tyr705) in vastus lateralis muscle. The panel shows the cellular localization phospho-STAT3. While there is no or very few nuclei positive for phospho-STAT3 in control and LO samples, phospho-STAT3 is clearly localized in the nuclei of EO mice muscle. Representative images of controls, LO, and EO at 40x magnification. Phospho-STAT3 (Tyr705) = red; nuclei = DAPI.

After examining the phosphorylation status of STAT3, we investigated the cellular localization of pSTAT3 (Tyr705). Immunofluorescence staining using a pSTAT3 (Tyr705)-specific antibody showed that expression of pSTAT3 (Tyr705) was visible and was localized in nuclei of the muscles of the EO mice, but was not detectable in the LO mice and controls (Figure 2). Approximately 50% of nuclei in the vastus lateralis muscles of the EO mice were positive of pSTAT3 (Tyr705). When costained with PAX7, a satellite cell marker, no pSTAT3 positive cells were costained. Our previous studies showed no overt inflammatory infiltration in the muscles of these mice [5]; therefore, most of the positive nuclei are likely myonuclei. To confirm that, we costained muscle sections with CD14 and CD11b (monocyte/macrophage markers) and CD3 (lymphocytes marker), respectively. In the few positive cells, no nuclei were costained with pSTAT3 (Tyr705).

### 3.3. TGF*β*1 Induces Tyr705 Phosphorylation of STAT3 in C2C12 Myoblasts

**Figure 3:**
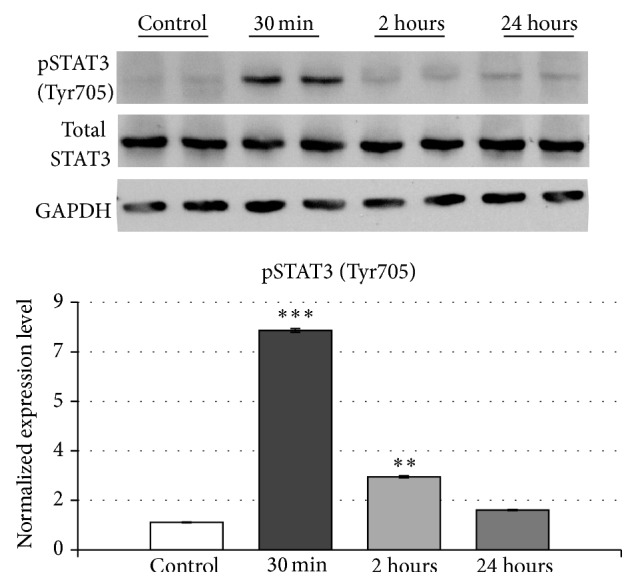
STAT3 phosphorylation level in differentiated C2C12. The panel shows STAT3 phosphorylation in differentiated C2C12 after TGF*β*1 10 ng/mL treatment. Protein extracted from C2C12 in control conditions, after 30 minutes, 2 hours, and 24 hours after TGF*β*1 10 ng/mL treatment, with anti-phospho-STAT3 (Tyr705), anti-total STAT3, and anti-GAPDH. The graph shows fold changes of phospho-STAT3 (Tyr705) normalized to GAPDH ± SEM. ∗∗ indicates *p* < 0.01; ∗∗∗ indicates *p* < 0.001.

In order to determine whether TGF*β*1 can directly activate STAT3 in muscle cells, we treated the murine myogenic cell line, C2C12, with recombinant TGF*β*1 protein. The pSTAT3 (Tyr705) level was determined by immunoblotting. The results showed that TGF*β*1 significantly increased pSTAT3 (Tyr705) 30 minutes after the treatment (7.9-fold, *p* < 0.001). The pSTAT3 (Tyr705) level descended to baseline level after 2 hours of treatment. There was no change of the total STAT3 protein during the time course examined between the treated and control groups (Figure 3).

## 4. Discussion

In this study, we explored the relationship between TGF*β*1 and STAT3 activation using a tet-repressible muscle-specific TGF*β*1 transgenic mouse and C2C12 cells. The phosphorylation of the Tyr705 residue is required for STAT3 dimerization, nuclear translocation, and DNA binding [28, 29]. Phosphorylation of the Ser727 residue is believed to promote STAT3 transcriptional activity through the enhanced recruitment of transcriptional cofactors, which is not required for functional activation of STAT3. Our data showed that overexpression of TGF*β*1 increased the amount of pSTAT3 (Tyr705) significantly in the EO mice but not in the LO mice. In addition, the effect was mediated through the phosphorylation of Tyr705 but not Ser727. While TGF*β*1 mediated activation of STAT3 in skeletal muscles was not reported previously, TGF*β*1 activated STAT3 by phosphorylating Tyr705 has been reported in hepatic cells and a mouse model of hepatocellular carcinoma [27, 30]. Both of these studies reported a direct activation of STAT3 by TGF*β*1. Interestingly, a recent study showed that hepatitis C virus (HCV) activates TGF*β*1 expression via STAT3 in hepatic stellate cells [31]. These findings suggested a potential postitive feedback loop between TGF*β*1 and STAT3 in the hepatic cells. In our TGF*β*1 transgenic model, we previously reported that expression of endogenous TGF*β*1 was induced in mice with more severe phenotypes, suggesting potential involvement of a positive feedback loop [5]. Whether the STAT3 activation directly modulates genes involved in muscle atrophy and fibrosis as reported in previous studies or it induces endogenous TGF*β*1 expression which is responsible for more severe phenotypes needs to be further examined.

STAT3 was originally reported for its capacity to mediate signaling predominantly from cytokines such as IL-6, IL-11, leukemia inhibitory factor (LIF), and oncostatin M. It is expressed in a large number of tissues and its activation drives the transcription of genes encoding proteins involved in angiogenesis, inflammation, apoptosis, extracellular matrix deposition, and cellular signaling [32]. IL-6 is well known for its crucial role in maintenance of skeletal muscle metabolism [33–35]. IL-6-induced STAT3 has been shown to promote satellite cells proliferation and myoblasts differentiation. Acute and transient activation of STAT3 via Tyr705 phosphorylation by IL-6 was reported to be associated with muscle hypertrophy after 10 weeks of resistance training in rats [36]. The hypertrophic effect was associated with the early upregulation of the IL-6/STAT3 signaling pathway and the downregulation of myogenic regulatory factors, including Pax7, MyoD, Myf5, and myogenin, in the satellite cells. While well controlled IL-6 expression plays a critical role in maintaining the homeostasis of skeletal muscles, studies also showed that persistent Tyr705 phosphorylation is associated with impairment of metabolism by negatively affecting skeletal muscle insulin signaling and glucose uptake [37] and is believed to be responsible for the IL-6-induced cancer cachexia [33, 38]. Our study showed that overexpression of TGF*β*1 for 2 weeks induced pSTAT3 (Tyr705) in skeletal muscles of the mice with severe phenotypes. Approximately half of the nuclei were positive for pSTAT3 (Tyr705). Since TGF*β*1 was the only gene overexpressed in the mouse model and is the driving force of the disease phenotype [5], the data suggested that TGF*β*1 is able to activate the STAT3 signaling directly. However, whether IL-6 signaling is involved in the process is not clear and needs further investigation. To examine the possibility and demonstrate that TGF*β*1 directly activates STAT3 in skeletal muscle cells, we treated the C2C12 cells with recombinant TGF*β*1 and examined the activation of STAT3 at 3 time points (30 min, 2 hrs, and 24 hrs) within 24 hours. Our findings showed that the pSTAT3 Tyr705 was transiently activated within 30 minutes. This result is in agreement with a recent study using immortalized rat hepatic stellate cells (HST) [27]. In this study, it was also shown that JAK1 is necessary for the Tyr705 phosphorylation and activation of STAT3. Knockdown of JAK1 but not JAK2 or Tyk2 is sufficient to attenuate TGF*β*1 mediated STAT3 activation. STAT3 is canonically activated by JAKs (JAK1, JAK2, JAK3, and Tyk2), which in turn are activated by a large number of cytokine and growth factors, including IL-10, IL-6, and EGF, and it is well known to play a crucial role in myogenic proliferation and differentiation [39, 40]. Whether the activation of STAT3 is mediated through JAK1 in our models needs to be investigated further.

## 5. Conclusion

Our study demonstrated that pSTAT3 (Tyr705) activation is associated with severity of phenotypes of our mouse model overexpressing TGF*β*1 in skeletal muscles. The activated STAT3 was localized in the nuclei of myofibers, suggesting transcription activation. Both* in vivo* and* in vitro* data suggested that pSTAT3 (Tyr705) can be induced by TGF*β*1. These data thus point to a novel signaling pathway that may modulate and contribute to the molecular and cellular mechanism of skeletal muscle fibrosis and atrophy in various diseases.
